# Exploring Speech and Language Therapists’ Perspectives of Voice-Assisted Technology as a Tool for Dysarthria: Qualitative Study

**DOI:** 10.2196/75044

**Published:** 2025-09-02

**Authors:** Jodie Mills, Orla Duffy, Katy Pedlow, George Kernohan

**Affiliations:** 1 School of Health Sciences Faculty of Life and Health Sciences University of Ulster Belfast United Kingdom; 2 School of Nursing Faculty of Life and Health Sciences University of Ulster Belfast United Kingdom

**Keywords:** smart speakers, speech and language therapy, Parkinson disease, dysarthria, voice disorder

## Abstract

**Background:**

People living with Parkinson disease (PD) often experience low speech volume and reduced intelligibility. Research suggests that common voice-assisted technology (VAT) devices, like Amazon Alexa and Google Home, can encourage individuals to modify their speech, speaking more clearly, slowly, and loudly. This highlights the potential of VAT as a therapeutic clinical tool in speech and language therapy (SLT). However, while VAT is emerging as a novel health care technology, gaps exist regarding understanding speech and language therapists’ (SaLTs) experiences using these devices in clinical practice for PD-related speech and voice difficulties.

**Objective:**

This research set out to explore various experiences of using VAT to address hypokinetic dysarthria, secondary to PD, from a range of stakeholder perspectives. This paper specifically focuses on clinical insights from SaLTs.

**Methods:**

SaLTs with prior experience of using smart speakers in clinical practice with people with speech or voice difficulties were invited to participate in focus groups or interviews. Between September and December 2024, seven SaLTs participated in semistructured focus groups or interviews using a topic guide. Discussions were informed by published evidence. Results were transcribed and analyzed using a framework analysis approach and were managed through NVivo software (Lumivero).

**Results:**

Four main themes were identified across the groups: (1) potential for VAT in SLT, (2) managing therapeutic beige flags, (3) empowering SaLTs to become digitally enabled practitioners, and (4) envisioning the future of VAT in SLT.

**Conclusions:**

This study recognizes VAT’s potential as a therapeutic tool that may improve volume, clarity, intelligibility of speech, and facilitate at-home practice for people with PD. However, before VAT can be widely implemented, considerations around data privacy, device limitations, and practical integration into clinical care must be addressed. Future research is proposed to design solutions to address usability challenges for both clients and clinicians. Finally, this paper offers key clinical recommendations for the development of a therapeutic VAT tool for speech and voice difficulties in SLT.

## Introduction

Up to 90% of people with Parkinson disease (PD) develop hypokinetic dysarthria [1], characterized by masked facial expression, reduced range of motion and volume, imprecise articulation, accelerated speech rate, prosodic insufficiency, reduced stress, rapidly repeated phonemes, and, in some cases, palilalia [2]. These speech impairments worsen over time due to the progressive nature of PD, resulting in low volume, which contributes to poor intelligibility and clarity of speech. This can lead to embarrassment, reduced well-being, social participation, and a loss of identity [3,4].

Early speech and language therapy (SLT) intervention is recommended during the management of PD [5], with guidance suggesting that therapists use compensatory strategies, external aids (eg, pacing boards, voice amplifiers, and augmentative alternative communication devices), or evidence-based treatments. The gold-standard therapy for hypokinetic dysarthria is Lee Silverman Voice Treatment (LSVT) LOUD [6,7], which targets vocal loudness and recalibrates internal monitoring mechanisms, using principles of motor learning and neuroplasticity. This focuses on the cue “BE LOUD,” aiming to target volume, and often has subsequent positive implications for articulation. Therapy is comprised of four areas: (1) daily exercises including a sustained phonation, pitch glides and functional phrases, salient speech hierarchy tasks across words, phrases, sentences, and conversation; (2) recalibration using feedback tools and education around changes in speech in Parkinson; (3) daily homework exercises; and (4) carryover exercises to promote use of loud voice in everyday life. However, LSVT is resource-intensive, and four sessions each week, for a month, are recommended to promote lasting changes. Often, there is a shortfall between these evidence-based recommendations and the real dosage provided during face-to-face therapy. This limits the accessibility of LSVT in the United Kingdom. Furthermore, regional speech and language therapist (SaLT) shortages and postpandemic waiting lists exacerbate difficulties accessing SLT [8], leaving a large percentage of people with PD without therapy access. Given these barriers, alternative therapeutic approaches are required to facilitate increased access to SLT for people with PD.

Voice-assisted technology (VAT) uses natural language processing and automatic speech recognition (ASR) to interpret speech and translate it into actionable requests. These devices are consumer-facing apps of artificial intelligence, and help the devices to provide interactive, contextually aware responses. VAT is more commonly known by commercial names such as “Amazon Alexa” and “Google Home,” or as a smart speaker. VAT has potential as a therapeutic tool in SLT for people with speech and voice difficulties [9-11]. However, dysarthric speech is often misinterpreted by VAT, with recognition accuracy decreasing as speech severity worsens or sentence length increases [12,13]. Despite this, error feedback can prompt some participants with speech difficulties to adapt their speech to enable interaction with VAT [14,15]. For example, people with PD have reported increasing their vocal effort, loudness, and clarity when interacting with VAT [10]. This is similar to the aims of LSVT, and findings are supported by wider literature [9,11]. Additionally, some SaLTs have integrated VAT into therapy for individuals with dysarthria, finding that it provides objective and impartial feedback on speech clarity and fosters improvements in volume and intelligibility [16]. However, it is important to note that the outcomes in this literature are self-reported, perceptual changes in speech and voice, rather than objective, quantifiable changes. This highlights a gap within the literature regarding the acoustic outcomes associated with VAT use for speech and voice.

While research has not yet established functional outcomes of VAT use in clinical practice, research indicates potential positive functional implications. VAT can create communication opportunities with others in the household [14,17] and contributes to increased speech confidence [10,16]. This is because VAT is perceived to provide objective, unjudgmental feedback on speech, which can reduce social barriers for users and the anxiety and embarrassment associated with repeating themselves [9,10]. Furthermore, the everyday nature of VAT avoids the stigma associated with bespoke SLT communication tools [10,16]. Notably, research also indicates potential functional risks when using VAT: communication avoidance due to increased awareness of difficulties [9] and frustration with speech not being recognized and subsequent error recovery [10]. VAT has also been recognized as a motivational tool for encouraging home-based SLT practice, addressing a recognized limitation of LSVT, where practice is described as repetitive and lacking real-world validity [18]. However, despite a growing evidence base for the use of VAT as an emerging therapeutic technology in SLT, to date, there is limited exploration of SaLTs’ experiences of using VAT therapeutically with people with speech and voice difficulties. Understanding SaLTs’ experiences of VAT ensures that future developments can meet their clinical needs and may reduce technology abandonment [19].

Therefore, this study aimed to explore the experiences of using VAT to address hypokinetic dysarthria, secondary to PD, from the perspectives of SaLTs.

The objectives of this study are as follows: (1) to understand how SaLTs are using VAT to support those with dysarthria; (2) to explore the therapeutic potential of using VAT for speech and voice outcomes, from SaLTs’ perspectives; and (3) to establish how to support the implementation of VAT in SLT.

## Methods

### Study Design

A qualitative methodology using online focus groups and individual interviews was used to explore SaLTs’ experience. Both SaLTs and researchers participated online via videoconferencing in their own respective homes, and no nonparticipants were present. Engaging in focus group discussions fosters dynamic conversations, often leading to fresh insights and idea generation [20], which is particularly valuable when evaluating the use of emerging technology. Notably, online focus groups have been found to produce a greater variety of novel ideas compared to traditional in-person discussions [21], as participants are described to be more “professional,” taking time to listen and consider their contributions before responding to questions or suggestions during online interviews and focus groups.

Individual interviews were used to supplement focus groups due to SaLTs’ scheduling conflicts. Online videoconferencing software (Teams; Microsoft Corp) was used, enabling data collection across a wider geographical area [22]. The lead author is a SaLT who has current and ongoing experience with the Parkinson population. It is acknowledged that this position could lead to potential bias in the interpretation of findings. Several measures were taken to address this, including transcription of focus groups and interviews within 24 hours and the use of a reflexive diary, which are further discussed within the data analysis section.

### Ethical Considerations

This study was reported in accordance with the COREQ (Consolidated Criteria for Reporting Qualitative Research) checklist [23] and is part of a larger PhD study that received ethical approval from the Ulster University Research Ethics Committee in July 2024 (FCNUR-24-016-A). All participants provided informed consent. Privacy and confidentiality were ensured: only the research team was able to see personal demographic data, and data will only be held as long as the university guidelines and General Data Protection Regulation (GDPR) require. Following transcription, all participants’ data were anonymized. Participants were reminded that if they knew another participant at the focus groups, they were to respect their right to confidentiality in their participation. No participants in the research were given payment.

### Patient and Public Involvement

A patient and public involvement steering group was established prior to study commencement to provide a voice for key stakeholders and ensure they played an active role in shaping the research. This group included a SaLT with firsthand experience using VAT in clinical settings, a person living with PD, and a caregiver. These three “experts by experience” helped to co-design the project, influencing and informing this work’s aims, methodology, and dissemination. Experts by experience were asked to provide feedback on information sheets via email, indicating that complex language should be avoided. Consequently, information sheets were revised with more patient-facing language. Furthermore, feedback on the interview schedule was also provided, with experts by experience suggesting that focus groups should build in time for participants to discuss their personal or professional journeys. They also suggested that introductions from researchers should include an informal preamble about smart speakers, which emphasized the technology’s relevance for people with Parkinson and the SaLTs working with this population. As a result, focus groups began with a summary of professional journeys, gave background to what smart speakers are, why they might work to improve speech and voice, and the lead author’s experiences of using her own personal “Alexa” device. Experts by experience also offered valuable insights of lived experience to the academic team during data collection and charting, including suggestions to offer the option of individual interviews to SLTs who could not attend focus groups, to maximize data collection.

### Recruitment

SaLTs were recruited purposively in the United Kingdom through the Royal College of Speech and Language Therapists, including the Parkinson’s Clinical Excellence Network and Bulletin Magazine, to participate in online focus groups and interviews. The anticipated sample size was difficult to determine without concrete and up-to-date figures of SaLTs working with adults in the United Kingdom. This study had a smaller number of participants than the suggested six to eight in the literature [24] due to pragmatic considerations and challenges with recruitment and scheduling. To balance diverse perspectives with data saturation, a range of 2-4 focus groups was proposed. Ultimately, two focus groups and two individual interviews were conducted.

Participants were asked to express their interest in the study, and following initial contact, they were screened via email or phone, according to study inclusion criteria, by the lead researcher (JM). Participants were unknown to the research team before participating. Eligible SaLTs were required to be older than 21 years of age, as this is the earliest age at which clinicians can be qualified, and were required to have prior experience of using VAT in clinical practice, with individuals experiencing dysarthria. It should be noted that four of the seven SaLTs directly worked with people with PD, and the others with populations experiencing dysarthria. Additionally, participants required access to an internet-enabled device with a camera for videoconferencing. There was no minimum clinical practice experience or experience of using VAT in clinical practice required to participate. Given that VAT use in the United Kingdom is still deemed to be emerging, this is a small, niche population. Increased inclusion criteria may have further limited the sample size. SaLTs meeting the inclusion criteria received an information sheet and consent form via email. Once consent was obtained, participants completed a digital health readiness questionnaire (Multimedia Appendix 1) and a demographic survey (Table 1). They then provided their availability for scheduling focus groups. SaLTs unable to join a focus group were offered the option of an individual interview. No background information was gathered relating to the severity of dysarthria of clients the SaLTs worked with, due to the exploratory nature of the study. Additional research may seek to investigate this further.

**Table 1:** Demographic data for SaLT^a^ participants.

SaLT ID	Gender	Age (years)	Clinical experience	Current clinical population	Location
P1	Female	46	23 years—specifically Parkinson	PD^b^	England
P2	Female	33	12 years	Adult disability or TBI^c^	England
P3	Female	41	11 years—specifically Parkinson	Adult community—Parkinson, MND^d^, MS^e^	Scotland
P4	Female	24	6 months	Adult community, including Parkinson	England
P5	Female	36	2 years	Adult community, including Parkinson	England
P6	Female	38	17 years—5 years of acute experience with Parkinson	TBI	Northern Ireland
P7	Female	43	18 years—specifically Parkinson	TBI	England

^a^SaLT: speech and language therapist.

^b^PD: Parkinson disease.

^c^TBI: traumatic brain injury.

^d^MND: motor neuron disease.

^e^MS: multiple sclerosis.

### Procedure

A total of 7 SaLTs were recruited and participated in either a focus group (n=2 groups) or an individual interview (n=2 interviews). No participants dropped out of the study. The sample size and duration of each focus group or interview are shown in Table 2.

This study was conducted as part of a larger study with people with PD, carers, and SaLTs, participating in six focus groups and two interviews. This paper only reports findings from seven SaLTs, and findings from focus groups with people with PD and carers are presented in another paper [25].

Data collection was facilitated online by the lead author (JM), a female SaLT, and a coresearcher (a qualified SaLT or health care professional with a PhD), and lasted between 38 minutes and 1 hour 30 minutes. The lead author participated in training on leading focus groups prior to conducting this research. To ensure methodological consistency, a semistructured topic guide (Multimedia Appendix 2), informed by literature, was used, and SaLTs had access to this in advance of their focus group or interview. This included prompting questions to encourage reflection and elaboration by clinicians and was reviewed by the experts with experience before use. Participants were invited to share their experiences of using VAT as a tool to address speech and voice difficulties with adults, and field notes were made during the focus groups or interviews.

**Table 2:** Format of qualitative data collection and number of participants for each.

Format	Number of participants	Length of time
Online focus group 1	3	1 hour 30 minutes
Online focus group 1	2	1 hour 7 minutes
Online interview 1	1	38 minutes
Online interview 2	1	1 hour

### Data Analysis

Focus groups were recorded audio-visually using Teams and were transcribed by the lead author (JM). To ensure methodological rigor and to ensure the accuracy of contextual memory, transcriptions of videos were completed within 24 hours of the focus groups. Before analysis, transcripts were anonymized, removing personally identifiable details, to ensure confidentiality.

Framework analysis was used to synthesize the results, as it facilitated understanding of results across a wider dataset [26]. Given the lead researcher’s background in SLT, framework analysis also supported reflexivity, encouraging critical reflection on biases and assumptions to promote analytical rigor [27]. The analysis followed the five-step process outlined by Ward et al [28]: (1) data familiarization, (2) framework development, (3) systematic coding, (4) synthesis within an analytical structure, and (5) interpretation.

The initial theoretical framework was created using published VAT evidence [13,16]. Once transcriptions were completed, data were imported into NVivo (version 14; Lumivero) for systematic organization and qualitative analysis. The researcher engaged in repeated readings of the transcripts, using mind mapping and a reflexive journal to document emerging themes. Preliminary themes were noted and discussed with coauthors (OD, KP, and GK). This led to further data immersion and refinement by the lead author (JM) before creation of draft themes. A consensus was reached by the team following further reflection on the data. The finalized framework was developed by systematically indexing data, summarizing this into codes, and charting findings into a structured analytical model [26]. Direct participant quotations are presented to provide context, to link data with interpretations, and to enhance the credibility of findings. Furthermore, results were sent to all participants for member checking to ensure written findings reflected their lived experiences and to enhance the rigor of the research.

## Results

### Overview

The Digital Health Readiness questionnaire required participants to rank 20 statements about technology use and ability, using a 5-point Likert scale. Statements covered digital access, use of digital technology, digital literacy, digital health literacy, and learnability.

Although seven SaLTs were recruited, 1 (P5) did not return the digital health readiness questionnaire. All 6 SaLTs who responded had good digital access: they all used the internet, a laptop, and a smartphone or tablet daily, 5 used health-related apps, and all indicated digital health literacy. All were motivated to learn about technology and strongly agreed that they would learn quickly when offered personal guidance about digital technology. Multimedia Appendix 2 provides further details.

There were four main themes identified across the groups and datasets: (1) VAT as a potential driver of positive speech therapy outcomes, (2) managing therapeutic beige flags, (3) empowering SaLTs to become digitally enabled practitioners, and (4) future design and features of a VAT tool (Textbox 1).

**Results from research with themes and subthemes.**

**Theme 1: Voice-assisted technology (VAT) as a potential driver of positive speech therapy outcomes**

1.1 Enabling speech practice and self-management

1.2 VAT reduces practice stigma as an everyday device

**Theme 2: Managing therapeutic beige flags when repurposing commercial technologies**

2.1 Usability barriers to therapeutic benefit

2.2 Need for monitoring and human interaction

**Theme 3: Empowering speech and language therapists to become digitally enabled practitioners **

3.1 Limited device knowledge for therapy

3.2 Suggestions for education and guidance

3.3 Navigating governance, data storage, and confidentiality

3.4 Smart speaker implementation concerns

**Theme 4: Future design and features of a VAT tool**

4.1 Develop a program or skill 

4.2 Lee Silverman Voice Treatment through VAT 

4.3 Additional feedback for self-awareness 

### Theme 1: VAT as a Potential Driver of Positive Speech Therapy Outcomes

Uses of VAT for managing dysarthria were reported by SaLTs, including therapeutic uses for speech and voice, VAT-supported self-management, and perceptions of VAT as an everyday, nondisability device.

#### 1.1 Enabling Speech Practice and Self-Management

SaLTs discussed the role of VAT in therapy and supporting home practice for carryover, with most considering VAT to be functional as part of their toolkit:

It’s used as a tool, as an adjunct ... It comes part of a massive part of treatment.

P7

I would almost separate it into two different roles ... The input would come from the therapy and the practice would come from the Alexa.

P5

You have all these like little homework tasks to do with LSVT. Alexa is kind of being your homework buddy, like a virtual assistant homework buddy for voice work.

P7

VAT was also identified as a practice tool for integrating volume, articulation, rate, and general intelligibility strategies:

I just ask them to use [it] in conjunction with strategies. So, we’ve got strategies we call SLOP strategies so slow, loud, over pronounce pause and making sure you take that deep breath.

P4

It’s like the whole needing to, remembering to breathe, activating it and then breathing again quickly and then really, really clear speech. So, it’s been a really good tool for him to try and remember to articulate the words quite clearly.

P2

SaLTs also indicated that VAT provided feedback on volume, clarity, and intelligibility of speech, and helped to develop self-awareness. Feedback can be a light on the device, or simply the device not responding to speech. This could prompt clients with dysarthria to adapt their speech using strategies:

They could be using it ... and getting that feedback themselves and thinking why is Alexa not understanding me. You know, if I raise my voice, will that help? If I be clear, will that help?

P6

So, he would ask, quite detailed biology questions ... then he would receive feedback if Siri understood him first time or not. It was, primarily for intelligibility feedback.

P6

It’s an alert for your kind of clear speech strategies: to speak louder, to go slower.

P7

SaLTs also explained that this feedback, including how well clients were understood by the VAT device or how well it completed their functional requests, was often central to their goals:

The other goal was for her to get feedback on her intelligibility ... So, when Alexa didn’t understand her, she had to repeat it and try and reduce the amount of time she would have to repeat it.

P6

With the speakers, you know, just being understood ... That’s quite a quick good way of monitoring their volume and their clarity I suppose if the speaker can understand them the first time.

P3

I’ll probably kind of set clearer goals within that report and say that we had a goal to kind of use Alexa, to be able to wake Alexa and kind of ask Alexa various questions and for that to work 70% of the time.

P7

 This objective and unjudgmental feedback from VAT devices was seen as a factor in encouraging the use of adaptive speech strategies and reportedly reduced the client’s reluctance to speak out:

We call it high challenge, low threat. Those activities that people ... they really want to have a go at, but that fear of failure, that fear of social rejection, isolation is taken away. Because the Alexa’s, you know, it’s going to respond to neither it’ll tell you “Oh sorry I didn’t understand that” or it will give you what, what you want.

P5

She really liked the idea of practicing with the Alexa to build her confidence.

P6

SaLTs also reported that VAT could be used to integrate functional phrase practice in the context of everyday requests whilst also promoting an increase in practice intensity for working-age clients:

Speech sounds on cue, or something like that ... they’re repeating random sentences that mean nothing to them. Whereas using Voice Assistant Technology they can ask whatever they want and get an actual outcome.

P6

We then developed kind of ten phrases that he could use for Alexa, to kind of say to Alexa and get Alexa to do various things ... So it’d be things like Alexa, what’s the weather today, Alexa, what time it?

P7

If people are working ... I think the intensity and the dosage would be good as well if they had access to that on a smart speaker and it would kind of do the role that we wouldn’t be able to offer.

P4

Furthermore, SaLTs reported that VAT could act as a communication partner for people with dysarthria:

I always go back to the means, reasons and opportunities model ... It’s when they don’t really have the opportunities to interact that often face-to-face with people, I tend to use that as their interaction opportunity.

P1

 Although this research focused on VAT as a therapeutic tool for speech and voice rather than a tool for environmental control, SaLTs indicated that VAT devices were often used for accessibility and environmental control, including clients interacting with VAT via augmentative alternative communication devices. The following quotes see SaLTs draw on wider experiences of working with clients with dysarthria, who did not have Parkinson:

He now has that set up at home in terms of turning on his lights and TV and things.

P2

They can use their eye gaze or their touch and have the computer speak on their behalf, and then it can turn on the lights or it’s connected to a smart plug so they can turn on their kettle to make a cup of tea.

P7

 Furthermore, by facilitating self-management of speech and voice difficulties, VAT could reduce reliance on ongoing therapy input and transfer responsibilities onto patients themselves:

Our aim is to deliver the strategies to people to self-manage, which, again is something that an Alexa could really help with ... It’s kind of moving patients away from that almost dependency on therapy and how can I self-manage.

P5

SaLTs also considered how VAT could be combined with SaLT-made resources to maintain speech and voice prior to SaLT, and following discharge:

We could always post it out to people as soon as they come on the waiting list and say, you know, if you’ve got a smart speaker. It’s worth a try. Just to maintain your functionality.

P5

Post therapy yeah, definitely. You could use it as a kind of maintenance tool to keep your kind of dysarthria strategies up.

P6

#### 1.2 VAT Reduces Practice Stigma, as an Everyday Device

SaLTs discussed the nature of VAT devices, which meant that they were widely integrated in households, and clients were motivated to practice with them. This also brought about a societal acceptance that VAT devices may reduce the stigma of using a speech-aiding device:

It is something that they’re familiar with and it’s in their home ... They are really excited to try and give that a go.

P4

It’s *not like a specialist piece equipment.* [P2]

They’re normal to integrate as part of your life, it’s much more acceptable ... It’s not like you’re singling them out and giving them some specialist equipment that doesn’t look or feel like the general population ... My clients often they hate to be reminded of their disability. They’re desperately trying to kind of live the life they lived before.

P7

### Theme 2: Managing Therapeutic Beige Flags

#### Overview

Participants reported several factors that must be considered before VAT can be implemented as a tool for managing dysarthria, including therapeutic and practical concerns and challenges posed by VAT devices. These considerations are termed beige flags, unlike red flags, as the barriers do not prevent the use of therapeutic VAT but can make it more difficult.

#### 2.1 Usability Barriers to Therapeutic Benefit

VAT devices not understanding clients, frustration, difficulty with error scaffolding, and devices timing out were reported as challenges when using VAT, although they did not necessarily preclude use.

Clients becoming frustrated when interacting with VAT was a concern for all the SaLTs:

At home, when we ask it something and then what it gives us is something completely random, it’s hilarious. But if you’ve got difficulty with speech intelligibility, I can imagine that’s not funny at all. It’s actually really frustrating and just another thing that confounds the fact that you’re struggling with your speech.

P1

I’m just aware of frustration as well: if they can’t access the Alexa and they are working so hard at it, depending on the severity of their dysarthria. It is important that it’s accessible to them and that they are getting success.

P3

SaLTs also explained that VAT often did not understand people, even when the clinician perceived their speech to be intelligible, and they highlighted how frustration could impact confidence. Additionally, it was recognized that fatigue and self-awareness could impact how well participants were understood by VAT:

It doesn’t matter how clear their speech is, sometimes the smart speakers just don’t get it ... It’s frustrating when my client sounds really clear to me but still isn’t picked up by the smart speaker. And trying to reassure them that it’s not them, it’s the speaker.

P7

Fatigue would also be an issue because they’re dysarthric. So, in the evenings, the girl who’s using it functionally would always say it’s harder to do it in the evenings.

P6

They’re not very good at changing their strategy, so they’ll just repeat the same sentence again and again.

P7

SaLTs also highlighted that additional factors, such as VAT timing out and difficulties with error scaffolding, hampered the therapeutic value of VAT. Error scaffolding related to feedback, both from the blue light on VAT devices that turns on when the device is listening, and the verbal feedback that devices may give when trying to action a request:

It didn’t actually give that feedback of “I didn’t understand that” the blue light just went off.

P4

When he is pausing, if it’s for too long, the device is stopping ... We’ve kind of had to start from scratch even though pausing is a really good strategy that I think is helpful in conversation.

P4

####  2.2 Need for Monitoring and Human Interaction

SaLTs discussed their reflections on therapeutic and practical concerns about using VAT in practice. Most clinicians indicated that they were unable to monitor clients’ use of VAT during therapy and following discharge:

I don’t want them to be unsuccessful either, and I’m not there to monitor that. I can’t see what happens at home.

P3

Another SaLT indicated that monitoring required extra support or additional staff:

I’ve been relying on either myself going in and monitoring that, a speech therapy support worker or, know a spouse or a carer of some sort.

P1

 Furthermore, VAT’s challenges in providing conversation, with standard settings, were considered as a barrier for the delivery of therapy. SaLTs indicated that VAT was not reflective of real-life communication challenges:

All we’ve been practicing is you ask a question; it gives a response. But we want to, you know, get back to conversations with people. And so, it’s kind of bridging that gap between these one-word responses to that device and how are you going to get back to the conversation?

P4

That sort of flow, the spontaneity and the flow of conversation is what patients need. It’s great for them to practice their speech with short questions and one-word answers, but actually that’s not what happens in communication and in conversation. It’s much more of a flow, and you do have to think on your feet.

P5

### Theme 3: Empowering SaLTs to Become Digitally Enabled Practitioners

#### Overview

Participants explained how a lack of adequate knowledge about what VAT can do, privacy, and practical and technical concerns prevented them from fully using the potential of the devices. They also suggested they needed guidance and education to help them further integrate VAT into clinical practice.

#### 3.1 Limited Device Knowledge for Therapy

 SaLTs discussed their uncertainties about what VAT can do, and all described a need for education about what Alexa can do:

I think it would be really interesting to know all of the other different services that a smart speaker offers, apart from just playing your favorite, your music or setting a timer.

P2

It depends on the therapist’s experience really with these smart speakers ... There’s those gaps as well if you’ve not used it in a specific way in your own home life, then you won’t know about it.

P3

SaLTs indicated that they required education and guidance about using VAT as a tool for dysarthria:

I think it would be really useful to have like a guidance booklet on kind of what a smart speaker is, how it’s used, like how the general population uses it, why people like it.

P7

#### 3.2 Suggestions for Education and Guidance

SaLTs also gave several suggestions about what they would like guidance and education to include. For example, resources, evidence-based practice, goal setting, and outcome measurement. Table 3 highlights and elaborates on these key ideas, using quotations.

**Table 3:** SaLT^a^ suggestions for education and guidance surrounding the therapeutic use of VAT^b^ for dysarthria therapy.

Suggestions for education and guidance	Supporting quotation
Resources and example therapy plans	“Even just like kind of therapy ideas pack. You know, this is what you could use it for. This is how you can use it, would be really nice because it’s hard when your kind of thinking about things to do sometimes.” [P6] “So, I think, I think so, just having those sorts of those ideas and even something that you know, you could, you could, you could print off for that that person as well. Anything that can just help generate ideas and then having the guidance is good.” [P3] “And then to have some ideas of things to try because I had to kind of look outside the speech therapy population for ideas on things to try with it ... I couldn’t come up with those on my own. So, I think just kind of showing people the different applications that you can use with smart speakers.” [P7] “Maybe also just kind of having like a little crash course into what people have found the best questions to ask are or some interesting ones that can prompt.” [P5]
Scripts	“So, we’re sort of saying the same sort of thing, to the patients. A wee script is always good.” [P3]
Idea sharing network with other clinicians	“Like a sharing of practice in terms of what works, what hasn’t worked, why hasn’t it worked? And is there, you know, is there something that we can do differently, better perhaps to, to get that?” [P5]
Goal setting	“How can you aim it to people who are working on different levels and different things? ... I think because everyone will use it in such a different way as well, depending on the severity of your dysarthria. And you know what, what your goals are as well. It would be really quite difficult currently.” [P4]
Outcome measurement	“Yeah, to measure that. What does progress look like? Because it will look very different for different patients depending.” [P5] “We’re always working on is strategies, it’s harder to sort of capture it when someone’s got a progressive condition, isn’t it?” [P3] “What about the sort of goal setting and evidencing, evidencing it in that sort of way? I think that’s always super, super useful. Not necessarily percentages, but if there was some sort of guidance.” [P3]
Evidence-based practice relating to dosage and maintenance	“I think it’d be useful in terms of knowing a bit of how much maybe you’d have to use it to improve speech ... I think there would be benefit from research to know, if you did this five times a day then your speech would increasingly get more intelligible.” [P2] “Evidence in terms of can they actually meet the levels of practice that you might need for maintenance, that would be something that I would find quite important.” [P4]
Evidence-based practice relating to therapeutic impacts	“If you’ve got that research behind it then you’ve got an easier sell to the rest of the team, and particularly with budget cut that we’ve had and the financial constraints, I think we do need this this sort of thing.” [P3] “If you were to do it purely from a data perspective, I wouldn’t be overly interested because we don’t work with data, we work with people ... if the data is telling me, yes, but the person is telling me no, then I’m not going to.” [P5] “I wouldn’t be overly worried about the evidence being like, really clear cut, I would be thinking instead, is this accessible to the patient? Is this something that they would find motivating? Do I think they would enjoy and benefit from it? Is it something that they would have to go out of their way to do?” [P5] “Because we don’t offer LSVT^c^, I think compared to another service that maybe do offer LSVT, they may want the research to be you know more developed.” [P4]

^a^SaLT: speech and language therapist.

^b^VAT: voice-assisted technology.

^c^LSVT: Lee Silverman Voice Treatment.

#### 3.3 Navigating Governance, Data Storage, and Confidentiality

SaLTs discussed common client concerns about privacy and their clinical reflections on this:

I think that is a concern with a lot of people, isn’t it? And it might be more of a concern with patients’ kids than patients themselves. You know, it would be ... Who’s listening?

P3

Maybe people being concerned about the device is always on and always listening to them ... How much information is it storing as well and what’s happening with that information.

P4

 One SaLT discussed how VAT’s recording of clients’ requests could be used in legal cases and described the measures she took to protect clients:

You can hear like domestic violence and things on the Alexa. People have kind of gone back and picked up arguments that have happened. You know, in police cases and thing ... I tried to make sure that when clients are kind of talking, their phrases are kind of pretty benign phrases and not giving away too much information.

P7

Furthermore, SaLTs indicated that GDPR and governance policies could act as barriers to VAT use. GDPR refers to guidance about how patients’ personal information is collected and stored, and is considered part of clinical governance. In the United Kingdom, the National Health Service has strict governance policies about how technology used in clinics must align with GDPR guidance:

We’ve had a lot of barriers from IT, from information governance ... IT security is a really, really big thing ... It’s all about GDPR.

P3

The huge amounts of red tape within the hospital trust just made it impossible.

P1

As a result of these concerns, SaLTs indicated that support was required:

I think [we need] all of the information that we just spoke about to do with risk information, governance, GDPR. All of those sort of guidelines around it.

P1

SaLTs also indicated that a clear, but realistic, view of risks and benefits from a clinical perspective would support the integration of VAT:

Information Governance is incredibly important, but I kind of feel like the risk benefit is skewed somehow? They need to be balancing it a lot better, whereas all they see is risk.

P1

We always chat to them about, you know, just about the information, keeping it safe and absolutely the benefit totally outweighs the risk. They’re like, I don’t care, like, I just want to communicate with people.

P3

####  3.4 Smart Speaker Implementation Concerns

Participants discussed technical and practical concerns about using VAT, as well as making suggestions for support.

VAT devices’ requirement for an internet connection meant SaLTs were unable to set up their own VAT for therapy, and therefore, most were only able to use VAT therapeutically if clients in the community owned a VAT device personally:

You can’t just take your smart speaker and plug it in in someone else’s house onto their Wi-Fi network, because immediately it knows it’s on a different Wi-Fi and it needs to set up an account and things.

P5

The barrier would be access, if the person has one or not ... I only use it when people have already got it set up.

P1

Neither myself or others are recommending they get smart speakers. We’re just going on the back of the fact that they already have them in the house and already using them.

P3

Furthermore, SaLTs expressed skepticism regarding VAT’s reliability for therapy and the unpredictability of responses. Variations in responses due to personalization and differences in plug-in features were also problematic:

I have no idea if this Alexa is going to answer me or not or what it’s going to say ... I can’t guarantee that it will continue to do that once I leave ... You know, it’s Harry Potter, isn’t it where they say, “Don’t trust something that can think for itself, if you don’t know where it keeps its brain.”

P5

“I don’t know what’s coming and that’s a difficulty for like therapy planning. It’s like you ask it to play some music and it says “you are not linked up to Amazon Music.” Well ... that’s a barrier because I don’t know what they have access to and what they don’t.

P4

Most SaLTs expressed a lack of clarity around SaLT roles when using VAT as a therapy tool. However, independent SaLTs had more role flexibility:

Thinking about our remit, the tech setup is not a speech therapy job! So again, that raises its own challenges.

P5

A lot of what we do is troubleshooting. So, we turn up at our client’s house, the Wi-Fi is down, and we’ve got time to call the Wi-Fi company or to switch the booster on or you know, that’s the way that we work. We’re really integrated into client’s homes.

P7

Given this, SaLTs explained that technology-specific guidance may help to overcome gaps in knowledge that were beyond clinical skills and would also help them with using VAT straight out of the box:

We’re speech therapists. We’re not technicians, we’re not clinical scientists. So, I think any support along those lines is, is always really useful.

P3

A set of instructions that you follow with your client in their house for the first set up, and then any other set up that they want to do, which might have to be done by someone else.

P5

Maybe also some tech support ... If it is just not responding to you, is there anything you can try?

P4

Furthermore, SaLTs also had practical worries about using VAT. A lack of consensus around how VAT is currently used therapeutically, dosage requirements, measurement of therapy outcomes, and maintenance or generalization was discussed:

I haven’t really done any sort of, anything formal with smart speakers.

P1

I don’t know ... I don’t give a particular dosage.

P1

Everyone’s doing something different in my team ... We’ve not been super specific in how we do it and actually in I suppose how we’re measuring our success as well.

P3

SaLTs indicated that future support may improve evidence-based practice using VAT, including demonstrating therapeutic impacts and required dosage to improve and maintain speech. Additional supporting quotes are found in Table 3.

It would be so exciting to strip everything away and just see if there’s parameters of speech and voice got better just on smart speaker treatment.

P7

I think I’d be really keen to hear about that more sort of qualitative ... the data that from that perspective, you know the stories of how is it helping, and is it helping, and to what extent is it helping, I think more so than the numbers.

P5

### Theme 4: Future Design and Features of a VAT Tool

#### Overview

Participants provided considerations for the future therapeutic use of VAT for people with speech and voice difficulties in SLT. This included developing VAT skills, delivering LSVT through VAT, increasing the ability to monitor speech and voice changes, and increasing levels of feedback to promote increased self-awareness.

#### 4.1 Develop a Program

Participants indicated that they would like VAT to have a program for speech and voice therapy. This was described in various terms:

I think it would be amazing if there was an Alexa skill that dealt with therapy.

P1

There are all sorts of apps that you can link to Alexa. So, there might be something where you could work on your pitch and Alexa would give you some feedback.

P7

#### 4.2 LSVT Through VAT

SaLTs also made suggestions regarding how a SLT VAT skill might function or features to include. For example, increased feedback, LSVT style exercises, conversation practice, and reminders to complete therapy tasks.

Participants described how LSVT-style exercises could be delivered through an Alexa skill, which could target articulation, breath support, phonation, singing, and reading:

I can see the 10 functional phrases. I can see that being practiced with a smart speaker. If you could think of things that you would say to Alexa where you get a response back where you know whether you’ve been clear enough, I can see that really working.

P7

SaLTs also suggested how reading aloud tasks could be facilitated by VAT:

You could get the lyrics to come down the screen ... could you do that with a reading passage? Could you get it to scroll a poem through in front of you?

P1

You could ask Alexa to listen to you whilst you read a passage for the kind of extra practice at the end.

P7

Furthermore, conversation was also highlighted as a beneficial feature for a VAT tool to incorporate:

I think just having those modelled conversations and actually having it answer you back with something that might be unexpected and you having to then formulate your response kind of like a conversation would be really helpful at the moment.

P4

 SaLTs felt these exercises could form part of home practice, and prompts and reminders could be useful to help clients integrate their everyday practice:

I’d use it for that, reminding them to do it as a routine. And then maybe you know a bit later ... “Have you done it yet?”

P1

####  4.3 Additional Feedback for Self-Awareness

 An enhanced feedback feature to facilitate self-awareness and recalibration of internal monitoring was suggested. More specific feedback from VAT on speech and voice was suggested. For example:

That wasn’t clear enough. Can you say it again? Louder.

P1

Oh, I didn’t quite understand what you said ... Could you slow down your speech?

P4

[VAT] could ... repeat back what they did understand

P3

 One SaLT indicated that this may help to overcome difficulties with communication partner prompting:

I find that quite a challenge to get communication partners to actually do that whole repeating back ... If a smart speaker could do that would be amazing!

P1

 Furthermore, SaLTs also indicated that scored feedback on intelligibility may be useful:

Sort of 6 weeks ago, I had to repeat myself 50% of the time, whereas now I’m only repeating myself 5% of the time.

P6

I mean, even better would be kind of being able to ... kind of give some feedback on percentage intelligibility or something, but I can imagine that would actually be very, very hard to develop.

P3

Finally, one SaLT suggested that this auditory feedback from VAT could be supplemented with visual displays and biofeedback:

If they’ve got that decibel meter in front of them and they could see that they’re hitting the red part of it. That’s the thing that helps ... I think some sort of biofeedback through a smart speaker would be really useful.

P7

## Discussion

### Principal Findings

This study aimed to explore SaLTs’ experiences of using VAT to address hypokinetic dysarthria. Focus groups and interviews indicated the potential of VAT for improving volume and intelligibility and supporting home practice. However, concerns about privacy, practical use, and device errors were noted. Key themes include VAT’s therapeutic use and potential, managing technical and practical challenges, supporting SaLTs to become digitally enabled using VAT, and the design and features of a future VAT tool.

### Therapeutic Potential as a Self-Management Tool

SaLTs discussed using VAT to support practice of volume, articulation, rate, and general intelligibility strategies, and reported using VAT as a biofeedback tool to provide feedback on speech clarity. This suggests that clinicians have experience of using feedback from VAT as an objective measure of intelligibility and to prompt increased self-awareness surrounding speech and voice. Kulkarni et al [16] reported similar findings, where SaLTs indicated that VAT could improve the accuracy of word productions for people with speech and voice disorders and increase clients’ motivation to practice at home. Indeed, several studies have indicated the potential for VAT as an SLT practice tool with participants, reporting a need for clear speech when interacting with the devices [12,14,29]. Furthermore, Smith et al [14] found improved speech intelligibility related to VAT use and device-related phrases and reported a smaller, but positive effect, for unrelated generalization phrases. This was echoed by Duffy et al [10], where people with PD reported speaking “slowly, loudly, and clearly” when using VAT. Findings from this study offer a foundation for collaboratively designing a VAT tool to support home practice and management of speech, voice, and self-awareness changes in neurodegenerative conditions.

Speech subsystems of articulation and phonation are commonly targeted by SaLTs during the therapeutic management of PD [30]. Although LSVT is the optimum, gold-standard therapy for people with PD [7], with clearly quantifiable improvements in volume [31], difficulties meeting intensity requirements, practice abandonment, and poor self-reported maintenance of therapy gains for people with PD can act as barriers [18,32]. VAT may have the potential to overcome these challenges by supporting the delivery of LSVT and facilitating home practice, shifting the focus from direct SLT input to self-management of longer-term conditions [33]. This is consistent with previous literature, which has suggested leveraging technology to facilitate practice of SaLT exercises and maintain therapy for people with PD or dysfluent speech [12,34]. Indeed, research has used technology at home following LSVT, known as LSVT Companion, and demonstrated gains in volume similar to in-person follow-up [35]. Literature reports that LSVT Companion can help to overcome constraints of treatment delivery, such as mobility and geographical constraints, while maintaining treatment efficacy, and may enable self-management of speech and voice difficulties for certain clients [35]. While Companion is specific to LSVT therapeutic tasks, it promotes digital use for therapeutic outcomes, particularly beneficial for providing biofeedback and outcome measures to reflect on the success of the tasks the clients have performed. Although Companion refers to laptop software, future research may consider how features of the LSVT Companion may be adapted and implemented by VAT.

However, SaLTs should be cautious that VAT is not used in isolation, but rather as a tool “to add to therapists’ toolboxes,” requiring SaLT-mediated support and check-ins. Current findings also overlap with wider literature regarding technology use in SLT (apps), where advantages such as increased practice frequency and intensity, alongside improved therapy motivation and engagement, are reported [36,37]. Given that improved vocal loudness, sensory recalibration, and intensive, high-effort practice are targets of traditional LSVT in SLT, future research may examine if and how VAT could host an alternative delivery of standard LSVT protocols or create “skills” for commercial VAT devices. Future research also may examine the quantitative impact of a VAT intervention to improve volume, clarity, and intelligibility for people with PD, using a combination of pre- and postmeasures and user self-reports.

Despite this, the technological caveats of VAT must be considered carefully by SaLTs. ASR errors are often higher than expected for dysarthric speech, and device inaccuracies may be attributed to ASR models and utterance complexities [38], as well as phonemic distortions associated with dysarthric speech. As discussed by SaLTs in this research, this creates concerns about a lack of consistency when using VAT for therapeutic tasks where there will be different devices, different rates of error, and even different environments. Furthermore, proper nouns, names, and locations may not be recognized by ASR [39]. Therefore, it may be difficult to use VAT to practice personalized, functional phrases, and it may limit use to basic interactional requests for some clients. Furthermore, the commercial technology exhibits an ethnocentric bias, with ethnicity and regional accents negatively influencing device recognition rates with commercial VAT systems like Apple, Amazon, and Google [40,41]. Additionally, high word error rates have also been found where individuals speak English as a second language [42]. SaLTs should therefore be mindful of attributing all device errors to clients, as other factors may be at play. Therapeutic use of VAT should ensure that users with speech and voice difficulties do not change nonstandard accents to more westernized accents to facilitate interactions with ASR [43], as this may perpetuate inequalities and racial bias at odds with person-centered practice in SLT.

### Balancing Therapeutic Considerations With Technical Challenges

SaLTs indicated that the use of VAT in SLT required careful balancing of facilitators and barriers during implementation.

SaLTs reported that device timing out, not understanding disordered speech, and a lack of meaningful feedback contributed to clients’ frustrations when using VAT. These difficulties were also reported in a recent scoping review examining the utilization of smart speakers by clients and staff in health care [33] and in wider literature [44-48]. Indeed, wider literature also indicates the impacts of using VAT on well-being. Decreased confidence, frustration with increased repetition, security anxieties, and avoidance of communication with smart speakers due to increased awareness of speech difficulties were noted [11-13]. Additionally, these error rates contributed to clinician perceptions that VAT was unpredictable, and SaLTs were hesitant to use VAT with participants who had more severe speech and voice difficulties, as they did not feel it would be beneficial. Literature examining SaLTs’ attitudes toward VAT reported the same clinical implementation concerns [15]. Similarly, SaLTs in Kulkarni et al [16] who had not used VAT, also perceived clients with severe speech impairment to be unable to use VAT effectively, with perceived negative implications for confidence and motivation.

Despite this, findings from this study also saw SaLTs report that VAT practice enabled clients to improve self-confidence and independence. Particularly, the everyday nature of VAT devices and acceptability had a motivational impact for clients. This suggests that VAT may be a socially acceptable tool for therapy, with the potential to reduce the stigma of practicing with an assistive device. This shows promise in reducing barriers to use and developing motivation to practice. VATs’ inclusivity as a therapy tool has been reported in literature, with studies indicating that VATs’ removal of social barriers and autonomous motivation may improve intelligibility [14] and contribute to feelings of inclusion for people with communication and cognitive differences [46]. This concurs with wider literature, which has also reported subjective improvements in confidence, self-awareness, accessibility, and well-being for clients when using VAT [45,48,49]. Increased independence, well-being, and quality of life for older adults and people with disabilities following VAT use were also shown [45,49,50].

Therefore, clinicians should exercise clinical judgment and balance risks against benefits on a case-by-case basis when implementing VAT with people with PD. While there is emerging evidence relating to VAT use with people with PD in SLT and general social benefits of VAT for older adults, much of the cited evidence is based on populations without neurodegenerative conditions. Subsequently, this represents a current gap in the literature, which this study begins to fill. VAT use is likely to impact people with PD differently due to the nature of the neurodegenerative disease, and prolonged unsupervised use of VAT could potentially have negative emotional consequences. Future research should explore VATs’ impact on motivation and therapy dosage in comparison to traditional SLT interventions.

### Support Needs

SaLTs indicated that they had gaps in their knowledge about VATs’ capabilities, as well as organizational barriers and privacy concerns. Therefore, participants highlighted that they would benefit from education and training, which would include technical education and reference material, privacy and security information, and therapeutic implementation guidance and resources. This aligns with previous research regarding the adoption and implementation of novel health care technologies, which frequently touches on privacy, GDPR, and confidentiality [13,51,52] and previous research surrounding the therapeutic use of VAT by SaLTs [16]. Future research must develop VAT standards specific to a health care context, establishing robust privacy and data security features with governance requirements in mind [33]. In the UK-based context, this should ensure VAT meets National Health Service GDPR collection and storage requirements.

While a lack of experience and training can contribute to frustration with technology [51], increased experience and knowledge can positively impact attitudes [53]. SaLTs’ opinions of information and communication technology in therapy were impacted by perceived performance outcomes, benefits to their work, and support in terms of education and training [54], and wider research indicates that promoting positive SaLT attitudes to technology and digital media is vital to supporting adoption [55]. It may be that increased knowledge of VAT’s capabilities in an applied SLT context could positively impact clinicians’ perceptions of VAT’s usefulness in SLT and lead to increased adoption. This is supported by technology adoption models such as the unified theory of acceptance and use of technology [56] and the technology acceptance model [57].

Despite this, no literature has examined education and training or knowledge translation interventions for clinical use of VAT as a tool for speech and voice difficulties. Literature regarding clinical applications of virtual reality in occupational therapy and SLT indicates that several education methods have been implemented, including manuals, web-based courses, and workshops [58,59]. Support for SaLTs using VAT with people with PD may include basic training about the range of technologies available, education regarding VATs everyday uses, skills and abilities, potential benefits, and information about applying the tool in practice, such as providing specific application examples with practice-based learning, information around privacy and GDPR, and linking to alternative delivery of known therapies such as LSVT [16]. Education and training strategies should work in tandem with research knowledge to translate the theoretical into practical and seek to empower SaLTs to become digitally enabled practitioners [60]. Future research should seek to create an educational VAT resource for SaLTs, using co-design methodology and behavior change frameworks to support the use of VAT as a therapeutic tool for the management of speech and voice disorders.

### Design and Features of a VAT Tool (A Vision for the Future)

SaLTs indicated that a future VAT tool for therapeutic management of dysarthria may take the form of an Alexa skill, with practice exercises for various speech subsystems, functional phrases, and conversation, as well as increased feedback on speech and intelligibility. Given that commercial VAT is a widely available technology, using available features to overcome the described limitations would facilitate increased accessibility and ease of use for SaLTs. Wider research has created physical activity and exercise interventions, based on recognized guidelines, and self-management instructions using Amazon Alexa Skills [17,61-63]. This indicates the potential for an Alexa Skill for SLT, which adapts standard LSVT protocols to facilitate alternative delivery and potential self-management as part of therapeutic management. Developer kits such as Alexa Blueprint and Alexa Skills Kits may be used. Research indicates that skills should be scalable, cost-effective, and customizable, with a focus on increasing motivation and engagement [33]. Alternatively, there may be skills available that can be repurposed for SLT purposes, and a voice assistant hub with suggested skills [24] may also be beneficial for SaLTs. Overall, to ensure VAT tools meet SaLTs’ clinical needs, future research should involve SLT end users in the cocreation of a VAT tool for the therapeutic management of dysarthria. This may use frameworks such as Design Thinking or ideas to focus on designing solutions to barriers suggested in this research, as well as ideating and refining suggested features.

### Limitations

This research was conducted with SaLTs based in the United Kingdom. Whilst participants work in a range of geographical locations across the United Kingdom, their experiences may not reflect the more diverse attitudes and experiences with VAT globally. Future work should replicate findings to ascertain result generalizability.

Second, to be eligible for participation, SaLTs had to be actively engaging with VAT already. These participants are likely to have positive attitudes to VAT and may have self-selected for participation. Furthermore, these SaLTs may also have adequate digital skills to use technology that may not have been evident in SaLTs who were not users of the technology. Given that the aims of the review focused on users of VAT, this is an expected limitation with the target population required to fully answer the research question.

Finally, due to scheduling conflicts, additional interviews to focus groups had to be completed. Given that there is a more transactional interaction during interviews, richer data may have been generated by the inclusion of these SaLTs in focus groups. However, this reflects the pragmatic considerations of conducting research with working clinicians and is a point of consideration for future studies.

Additionally, given the novel nature of the VAT technology, a low number of clinicians are actively implementing the technology in clinical practice. Therefore, this study had a smaller number of participants than the suggested 6-8 in the literature [24] due to pragmatic considerations and challenges with recruitment and scheduling. Therefore, individual interviews were completed with two SaLTs. As artificial intelligence will undoubtedly play a larger role in health care in the coming years, replication of this research in the future may yield interesting results.

### Recommendations

This study makes recommendations for future research to develop the therapeutic use of VAT:

- Co-design of a therapeutic VAT tool for hypokinetic dysarthria, which may include feasible solutions to technological limitations, to meet the clinical needs of SaLTs.
- Co-design of applied education and guidelines for therapeutic use of VAT, technology upskilling, and privacy and governance applications. Future work must also identify the optimal route for the delivery of education and training based on behavior change frameworks.
- A feasibility trial of a therapeutic VAT intervention for people with PD with dysarthria, including pre- and postacoustic analysis of voice or self-reported impacts.

### Conclusions

This study builds upon Kulkarni et al [16] by exploring SaLTs’ experiences of using VAT as a therapeutic tool for speech and voice difficulties. Positive therapeutic outcomes for intelligibility, volume, and clarity of speech are reported, with the potential for VAT to facilitate home practice and self-management of dysarthria. However, clinicians must balance this with well-being implications, device errors, practical implementation factors, and privacy concerns. These difficulties should be addressed in future research to enable the integration of VAT as a therapeutic tool.

Future research should use participatory research with clinicians using VAT to create achievable and practical solutions to VAT’s usability challenges and consider the adaptation of the standard LSVT protocol to facilitate the development of an Alexa Skill. The feasibility of a VAT tool for SLT may be established by adapting Smith et al [14]. By examining quantitative outcomes relating to intelligibility and volume and self-reported changes, as well as potential generalization and maintenance effects, an initial evidence base may be established for the use of this novel technology in SLT.

## Appendix

Multimedia Appendix 1 Showing the Digital Health Readiness Questionnaire results (number 1 in paper).

Multimedia Appendix 2 Discussion guide for focus groups and interviews.

## Abbreviations

ASR: automatic speech recognition
COREQ: Consolidated Criteria for Reporting Qualitative Research
GDPR: General Data Protection Regulation
LSVT: Lee Silverman Voice Treatment
PD: Parkinson disease
SaLT: speech and language therapist
SLT: speech and language therapy
VAT: voice-assisted technology
