# Supplementary material for: Exploring Speech and Language Therapists’ Perspectives of Voice-Assisted Technology as a Tool for Dysarthria: Qualitative Study
Source: JMIR Rehabil Assist Technol. 2025 Sep 2;12:e75044. doi: 10.2196/75044 (PMC12441644; doi:10.2196/75044)
Supplement: Multimedia Appendix 1 [file rehab_v12i1e75044_app1.docx]

| **Digital Access (people with Parkinson’s)** | I use the internet | I use a computer or laptop | I use a smartphone and or tablet | I use a wearable (fitness tracker, smartwatch etc) |
| --- | --- | --- | --- | --- |
| P1 | 5 - daily | 5 - daily | 5 - daily | 5 - daily |
| P2 | 5 - daily | 5 - daily | 5 - daily | 5 - daily |
| P3 | 5 - daily | 5 - daily | 5 - daily | 5 - daily |
| P4 | 2 - rarely | 5 - daily | 3 - sometimes | 1 - never / I don't have any wearable |
| P6 | 5 - daily | 4 - often | 5 - daily | 1 - never / I don't have any wearable |
| P7 | 3 - Sometimes | 5 - daily | 5 - daily | 5 - daily |

| **Usage of digital technology** | I am able to write and send an email independently | | I use social media | | | I am able to perform videocalling | | | I am able to take a picture and send it to another person | | | | I am able to register and review my daily step count | |  |
| --- | --- | --- | --- | --- | --- | --- | --- | --- | --- | --- | --- | --- | --- | --- | --- |
| P1 | 5 - strongly agree | | 5 - daily | | | 5 - strongly agree | | | 5 - strongly agree | | | | 5 - strongly agree | |  |
| P2 | 5 - strongly agree | | 5 - daily | | | 5 - strongly agree | | | 5 - strongly agree | | | | 5 - strongly agree | |  |
| P3 | 5 - strongly agree | | 4 - often | | | 5 - strongly agree | | | 5 - strongly agree | | | | 5 – strongly agree | |  |
| P4 | 5 - strongly agree | | 5 - daily | | | 5 - strongly agree | | | 5 - strongly agree | | | | 4 - agree | |  |
| P6 | 5 - strongly agree | | 3 - daily | | | 5 – strongly agree | | | 5 – strongly agree | | | | 5 – strongly agree | |  |
| P7 | 5 - strongly agree | | 5 - daily | | | 5 - strongly agree | | | 5 - strongly agree | | | | 5 - strongly agree | |  |
|  |  | |  | | |  | | |  | | | |  | |  |
|  |  | |  | | |  | | |  | | | |  | |  |
| **Digital Literacy** | I know how to find helpful and reliable information on the internet | | | | I feel safe when looking up information on the internet | | | | | | | I feel in control when looking up information on the internet | | |  |
| P1 | 4 - agree | | | | 4 - agree | | | | | | | 4 - agree | | |  |
| P2 | 5 - strongly agree | | | | 5 - strongly agree | | | | | | | 4- agree | | |  |
| P3 | 5 - strongly agree | | | | 5 - strongly agree | | | | | | | 5 - strongly agree | | |  |
| P4 | 5 – strongly agree | | | | 4 - agree | | | | | | | 5 – strongly agree | | |  |
| P6 | 5 - strongly agree | | | | 5 - strongly agree | | | | | | | 5 - strongly agree | | |  |
| P7 | 5 – strongly agree | | | | 5 – strongly agree | | | | | | | 5 – strongly agree | | |  |
|  |  |  | |  | | |  |  | |  |  | |  |  | |

| **Digital Health Literacy** | I use the internet to find more information about my symptoms, health status or medication | I use health-related applications to follow up my health status | I am able to identify trustworthy, reliable health information on the internet |
| --- | --- | --- | --- |
| P1 | 4 - agree | 4 - agree | 4 - agree |
| P2 | 4 - agree | 4 - agree | 4 - agree |
| P3 | 5 - strongly agree | 5 - strongly agree | 5 - strongly agree |
| P4 | 4 - agree | 2 - disagree | 5 – strongly agree |
| P6 | 5 - strongly agree | 5 - strongly agree | 5 - strongly agree |
| P7 | 5 – strongly agree | 5 – strongly agree | 5 – strongly agree |

| **Learnability** | I am motivated to learn more about digital technology and how to use it myself | I feel confident that I can learn about digital technology and how to use it myself | I believe that I will learn quickly when offered written information about digital technology | I believe that I will learn quickly when offered personal guidance about digital technology | I expect that learning digital skills can positively impact my health |
| --- | --- | --- | --- | --- | --- |
| P1 | 4 - agree | 5 - strongly agree | 4 - agree | 5 - strongly agree | 4 - agree |
| P2 | 5 – strongly agree | 5 – strongly agree | 4 - agree | 5 – strongly agree | 4 - agree |
| P3 | 5 –strongly agree | 5 – strongly agree | 5 – strongly agree | 5 – strongly agree | 5 - strongly agree |
| P4 | 4 - agree | 5 – strongly agree | 4 - agree | 5- strongly agree | 3 – neither agree nor disagree |
| P6 | 5 - strongly agree | 5 - strongly agree | 5 - strongly agree | 5 - strongly agree | 5 - strongly agree |
| P7 | 5 – strongly agree | 5 – strongly agree | 5 – strongly agree | 5 – strongly agree | 5 – strongly agree |
