# Supplementary figures and images for: Exploring Speech and Language Therapists’ Perspectives of Voice-Assisted Technology as a Tool for Dysarthria: Qualitative Study
Source: JMIR Rehabil Assist Technol. 2025 Sep 2;12:e75044. doi: 10.2196/75044 (PMC12441644; doi:10.2196/75044)

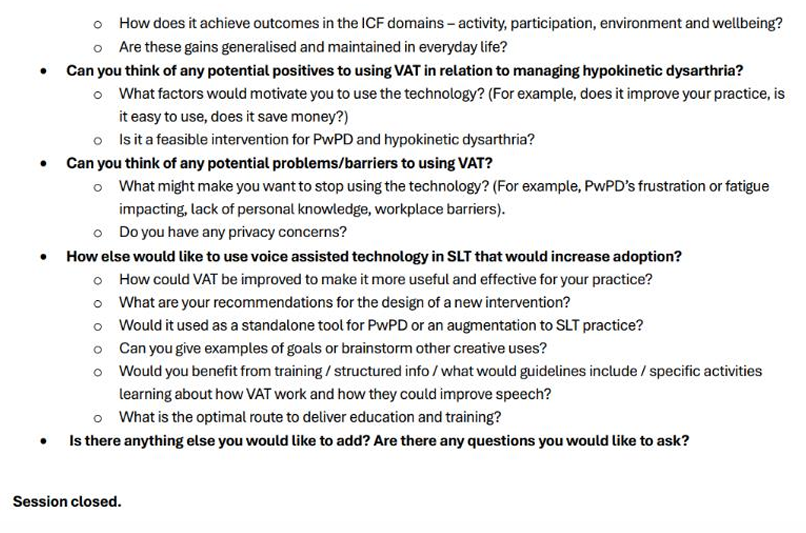

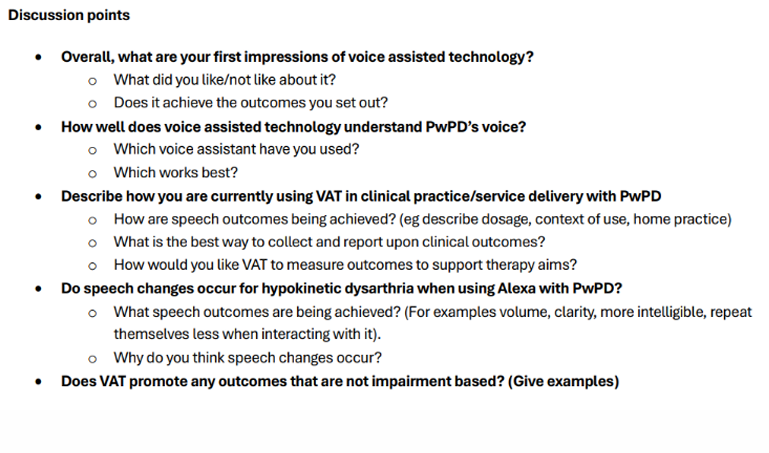

Supplement: Multimedia Appendix 2 [file rehab_v12i1e75044_app2.docx]
